# Current and Future Directions in Minority Aging: Embracing Interdisciplinary Models

**DOI:** 10.1093/geroni/igab046.368

**Published:** 2021-12-17

**Authors:** Heather Farmer, Amy Thierry, Keith Whitfield

## Abstract

Racial/ethnic disparities in health among older adults are well-documented. More research is needed to clarify the complex and multifactorial mechanisms underlying these associations. This symposium will feature research that employs innovative theoretical and methodological approaches to understand the biopsychosocial mechanisms that underlie racial/ethnic disparities in older adults’ health and determine sources of within-group heterogeneity in minority aging. Dr. Forrester will integrate stress biology and intersectionality to demonstrate the importance of stress and resilience (e.g., John Henryism) with biological aging within Black adults participating in the Coronary Artery Risk Development in Young Adults (CARDIA) Study. Dr. Brown Hughes will present innovative research using data from the African American United Memory and Aging Project (AA-UMAP) on the importance of Alzheimer’s disease-specific knowledge and perceptions among Black older adults. Dr. Gamaldo will employ a within-race approach to understand how knowledge and perceptions of Alzheimer’s disease and related dementias (ADRD) shape cognitive performance among Black older adults in the AA-UMAP study. Dr. Mitchell will use Health and Retirement Study data to explore the role of midlife stress exposure in accounting for racial disparities in trajectories of cognitive functioning. Drs. Thierry and Farmer will use HRS data to examine how psychosocial resilience (e.g., mastery) affects the relationship between perceived neighborhood conditions (e.g., disorder) and cognition among Black older adults. This work highlights the importance of applying an interdisciplinary lens to move the study of minority aging forward and ultimately, to reduce the unnecessary burden of morbidity and mortality among minoritized groups.

